# The development of a contextually appropriate measure of psychological distress in Sierra Leone

**DOI:** 10.1186/s40359-021-00610-w

**Published:** 2021-07-21

**Authors:** Rebecca Horn, Kanykey Jailobaeva, Stella Arakelyan, Alastair Ager

**Affiliations:** NIHR Global Health Research Unit On Health in Situations of Fragility, Institute for Global Health and Development, Queen Margaret University, Edinburgh, UK

**Keywords:** Scale development, Validity, Sierra Leone, Psychological distress, Mental health

## Abstract

**Background:**

Studies of psychological distress in Sierra Leone have typically used measures which were developed for use in other contexts, and which often have not been adapted or validated for use in Sierra Leone. This has resulted in a lack of reliable information about the patterns of psychological distress within the population, which is a barrier to the development of effective and appropriate mental health services. The aim of the study was to develop a locally-appropriate measure of psychological distress for Sierra Leone.

**Methods:**

The new measure consists of two instruments: the Sierra Leone Psychological Distress Scale (SLPDS) and a gendered measure of ability to carry out daily tasks—a Function scale—as an indication of the severity of distress. A three-phase mixed methods exploratory sequential study was conducted. Phase 1 was item generation and testing, leading to the development of a set of potential items for both instruments. Phase 2 was a small pilot study (N = 202) leading to the selection of the final set of items for both measures. Phase 3 was a validation phase where the SLPDS and the Function scale were administered with a larger sample of 904 respondents. Item analysis was used to assess the internal consistency of the scales, and Exploratory Factor Analysis to explore the properties of the SLPDS.

**Results:**

Exploratory factor analysis using the principal axis factoring with an oblique rotation identified a three-factor structure for the 18-item SLPDS. Internal consistency for the SLPDS (Cronbach’s alpha = 0.89) and three subscales was good (Cronbach’s alpha > 0.73). The internal reliability of the male and female versions of the Function scale was also found to be acceptable (Cronbach’s alpha = 0.90 for the female scale and 0.79 for the male scale).

**Conclusions:**

Together the SLPD and Function scales provide a locally-validated tool which will enable government bodies and local and international non-governmental organisations in Sierra Leone to assess mental health and psychosocial needs. This will support both effective service provision and the evaluation of initiatives designed to improve mental health and psychosocial wellbeing.

**Supplementary Information:**

The online version contains supplementary material available at 10.1186/s40359-021-00610-w.

## Background

Sierra Leone, in West Africa, experienced a brutal civil war between 1991 and 2002. An estimated 70,000 people were killed and more than 2 million (more than one-third of the population) displaced [1]. Following the war, efforts were made to rebuild systems and infrastructure within Sierra Leone, but these efforts were disrupted by the outbreak in 2014 of Ebola Virus Disease (EVD). This continued for almost two years and had a devastating effect on an already fragile population. Since March 2020, Sierra Leone, along with the rest of the world, has been dealing with the effects of the COVID-19 pandemic.

People in Sierra Leone have experienced multiple adverse events in the past, combined with current struggles to maintain wellbeing in one of the poorest countries in the world in terms of economic development, health, and education. Sierra Leone was ranked 182 out of 189 on the Human Development Index in 2020 [2].

The population of Sierra Leone has demonstrated remarkable resilience in the face of such a series of extreme events, but the coping capacities of individuals, communities and health systems have been severely challenged.

A National Mental Health Strategic Plan (2010–2015) and Mental Health Policy were developed in 2010, and, following the end of the EVD outbreak, an updated Mental Health Strategic Plan (2019–2023) was produced [3]. Priority issues include research on ‘culturally relevant mental health syndromes and descriptions’ which are ‘critical to guiding the implementation of evidence-based, culturally appropriate and efficient mental health services to meet the needs of Sierra Leoneans’ (p25). There is currently a lack of information on the forms of psychological distress experienced within the adult population of the country. Most published research on mental health in Sierra Leone relates to the impact of the prolonged civil conflict and the EVD outbreak [4, 5], with few studies focusing on mental health in the general population outside of an emergency context.

One of the barriers to gathering the information required to develop appropriate services and supports for those affected by mental health problems in Sierra Leone is that there is currently no locally-appropriate tool to measure psychological distress. Ventevogel and Faiz [6], amongst others [7, 8], have highlighted how the use of measures of mental health which have not been validated with the population with which they are used can create misleading findings, as they may give inflated estimates of distress as a result of conflating adaptive distress reactions with psychopathology. This has significant implications for the planning and provision of effective prevention and response services for those affected by mental health difficulties.

Some 35 years ago, Kleinman [9] suggested that local idioms of depression be translated and added to standard questionnaires, and subsequently, researchers have emphasised the importance of including locally salient concepts and phrases in tools to measure psychological distress and mental health disorders [10]. As a result, local assessment tools have been developed in various settings to enable more accurate assessments of psychological distress [11]. These local assessment instruments have been found to be more effective measures of mental disorders than international instruments in various populations [10, 12]. Studies have demonstrated how rapid ethnographic assessment can be used to develop locally-appropriate measures of psychological distress that are internally consistent [15], align with local understandings of distress [16], account for considerable variance in functional impairment [17], and can effectively evaluate interventions [18].

The availability of a locally-meaningful measure of psychological distress in Sierra Leone would enable government bodies (particularly the Ministry of Health and Sanitation and the Ministry of Social Welfare) and both international and local non-governmental organisations to assess mental health and psychosocial needs in order to plan effective service provision. Such a measure also has clear utility in the evaluation of programmes and initiatives to improve mental health and psychosocial wellbeing, so contributing to more effective programming and more targeted use of the limited funds available within this sector.

In this paper we describe the process of developing a tool to support such developments in Sierra Leone.

## Methods

The new measure consists of two instruments: a measure of psychological distress (referred to here as the Sierra Leone Psychological Distress Scale [SLPDS]) and a measure of ability to carry out daily tasks (referred to here as the Function scale). The second instrument is designed to be an indication of the severity of distress, as described by Bolton & Tang [16], reflecting gendered roles in Sierra Leone.

The methodology for the development of the tool drew on van Ommeren et al. [19] and others who have used similar approaches to develop locally-appropriate measures of psychological distress [20, 21]. We conducted a three-phase mixed methods exploratory sequential study. Phase 1 was item generation and testing, leading to the development of a set of potential items for both instruments. Phase 2 was a small pilot study (N = 202) leading to the selection of the final set of items for both measures. Phase 3 was a validation phase where the SLPD and the Function scales were administered to a larger representative sample of 904 respondents.

### Research team

The training and supervision of the research team was carried out by a Queen Margaret University researcher and coordination of logistical issues was conducted by a member of staff from the College of Medicine and Allied Health Sciences (COMAHS), University of Sierra Leone. The field researchers were all Sierra Leoneans, aged between 20 and 30 years old, and either recent university graduates or in the final phase of their studies. They were drawn from a range of ethnic groups, and spoke Mende, Temne, Fullah and Limba as well as being fluent in Krio and English.

A team of four field researchers (two female, two male) was involved in Phases 1 and 2, and a larger team of 10 field researchers (five female, five male) in Phase 3. The team took part in four days’ training for Phases 1 and 2, and five days’ training before Phase 3. The training consisted of sessions on research ethics, plus intensive practical training in the methods to be used. This included pilot testing and revision of the methodology.

### Phase 1: Development of SLPDS items and format

#### Selection of items for testing

The 30 signs of distress to be included in the tool were identified in an earlier qualitative phase of work which has been described elsewhere [22]. It was based on the ‘rapid ethnographic’ approach developed by Bolton and colleagues [15, 18] and included freelisting, key informant interviews and pile sorts.

Following this, instruments which had been used in Sierra Leone by other researchers were reviewed to identify items which could measure the 30 signs of distress. Where no existing items fitted a sign of distress, a new item was developed. In order to strengthen comprehensibility, items were framed as questions rather than statements [21] and the use of negatively worded items was avoided [23–28].

Six of the signs of distress identified by community members could not be reliably assessed through a self-report measure because they involved socially unacceptable behaviours and/or behaviours which somebody who is experiencing them is unlikely to have insight into. Items were not developed to assess these signs of distress.

Thirty-nine items were developed to reflect the remaining 24 signs of distress. More than one question was included for eleven signs of distress, with the aim of identifying the most effective items through the testing process.

#### Timeframe

The questions focused on experiences over the previous one week, based on the assumption that psychological distress would last with varying intensity for several days at least [29], and the fact that other widely-used measures of distress use this time frame (e.g. HSCL-25, Impact of Events Scale).

#### Response format

A four-point scale was used in an attempt to balance sensitivity with simplicity. Price, Conteh and Esliker [30], in their translation of the WHOQOL-BREF into Krio, found that some of the extreme anchor points in a five-point scale required the creation of terms that are not commonly used in Krio, raising questions of whether individuals are able to distinguish between the different options.

Instructions were ‘I will ask you about some difficult experiences that people sometimes have. I would like you to tell me how much you have had these experiences in the last one week, including today.’ They were then asked to choose one of the following options: Not at all; A little; Quite a lot; Very much.

**Fig. 1:**
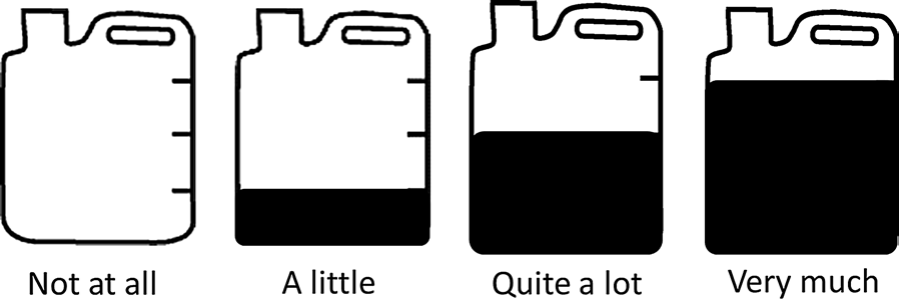
Illustrations of response options for psychological distress items

Given that visual illustrations can increase the comprehension of Likert scales (Betancourt, 2015), respondents were shown pictures of four containers, each with a different amount of water in to illustrate the different response options (see Fig. 1). They were told: ‘You can use the pictures of the jerry cans to help you if you like. The more water there is in the jerry can, the more you have had the experience over the last one week’.

#### Translation

The instructions and the 39 items were translated into Krio by a bilingual member of the research team who had participated in all stages of the project and therefore had aquired a good understanding of the meaning of the items and the purpose of the tool. The Krio version was back-translated into English by a bilingual person unfamiliar with the project.

#### First review of items

Five members of the research team (one British and four bilingual Sierra Leoneans), including the original translator, reviewed the 39 items. The review included the original English version of the items, the Krio translations and the back-translations. Challenges with particular items or words were discussed by the group and consensus reached on the best version of the Krio item to include in the testing process. In addition to reviewing the Krio version of the items, some other changes were made either because the original wording was confusing in the Sierra Leone context or because it was socially unacceptable. At the end of this process, there were 38 items included in the draft SLPDS.

#### Focus group discussions

Four focus group discussions (FGDs) were conducted in Freetown (two groups of men and two groups of women) and four FGDs were conducted in Bombali district (two groups of men and two groups of women). Participants were purposively selected to represent different ages and educational levels. Inclusion criteria were that participants must be 18 years or older, living in the province where the FGD is taking place and able to provide informed consent (i.e. no mental disability or serious developmental disorder). Participants were excluded from the study if they had a cognitive impairment which meant they were unable to give informed consent.

Each FGD consisted of eight participants (except one female group in Freetown, which consisted of nine participants), plus two facilitators. One facilitator managed the discussion while the other took notes and assisted with the facilitation when necessary. All discussions were conducted in Krio, and notes were made in a combination of English and Krio.

Participants were read each item in turn and asked: ‘what do you think this means?’ (to judge comprehensibility) and ‘how would you respond to this question? (to judge acceptability). The intended meaning of the item was then explained, and participants asked whether they have any suggestions as to how the item could be improved, such as different local words or ideas to communicate the idea being measured.

The research team subsequently reviewed the feedback on each item and t made changes to improve comprehensibility and acceptability. Following this process, the Krio wording of some items was changed, but the same 38 items were retained.

#### Cognitive interviewing

Cognitive interviewing, using the probing technique [31], was used to assess both the items and the process of completing the SLPDS. The process involved the interviewer reading each item on the questionnaire to the respondent and asking a series of questions about their understanding of that item.

Respondents were selected purposively by the field researchers to ensure that the items were tested with a diverse group of people. The same inclusion and exclusion criteria were used as for the FGD stage. Sixteen cognitive interviews were conducted with seven women and nine men. Ages ranged from 22 to 47 (mean age = 31.3 years, SD = 7.70), and people with a range of educational backgrounds were included. However, there was a higher proportion of people with tertiary education (7) than is representative of the Sierra Leone population. Six completed Senior Secondary School, one completed Junior Secondary School and two had no education.

Following the completion of the cognitive interviews, the research team met to review responses and make final revisions to the process and the items. A small number of revisions were made to the Krio version of the items during this process, and the order of the questions was revised so the more easily-answered questions came at the beginning and the end, with the more difficult ones in the middle.

### Phase 1: development of function scale

The development of the Function scale was based on the methodology developed by Bolton and Tang [16]. A freelisting exercise was conducted with a convenience sample of 94 community members (47 female and 47 male) aged between 18 and 70 (mean age = 33.2 years, SD = 12.26) in four districts of Sierra Leone (Western Area, Kambia, Kono and Bo) in order to learn about tasks important to local people. The respondents were asked to describe the normal tasks that women/men (depending on the gender of the respondent) were expected to do for themselves, their families and their communities. The interviewer probed to encourage the respondent to give as many tasks as they could think of. Once the respondent could not think of any more tasks, the interviewer then revisited each one they had listed and asked for a short description of each.

#### Selection of items for testing

The freelisting data were reviewed and all those that could not be considered as tasks were deleted (e.g. ‘women quarrel among themselves’, ‘women gossip’). The cleaned data was then categorised into three groups separately for men and women: self, family and community. The frequencies with which each task was identified were then calculated. Those which were most frequently mentioned were chosen for inclusion in the Function scale. The final version of the Function scale for testing included 10 items for women and 10 items for men, plus an item requesting respondents to identify any other important tasks. The translation of the items into Krio took place in the same way as for the SLPD scale items.

The template and instructions to respondents were based on those developed by Bolton and Tang (2002). Respondents were asked to consider each task read out to them and rate how much difficulty they had in doing it compared to most other men/ women of their age. Respondents were asked to state whether over the last one week they had had: no more difficulty than most other men/women of their age; a little more difficulty; a moderate amount more; a lot more; they cannot do the task.

**Fig. 2:**
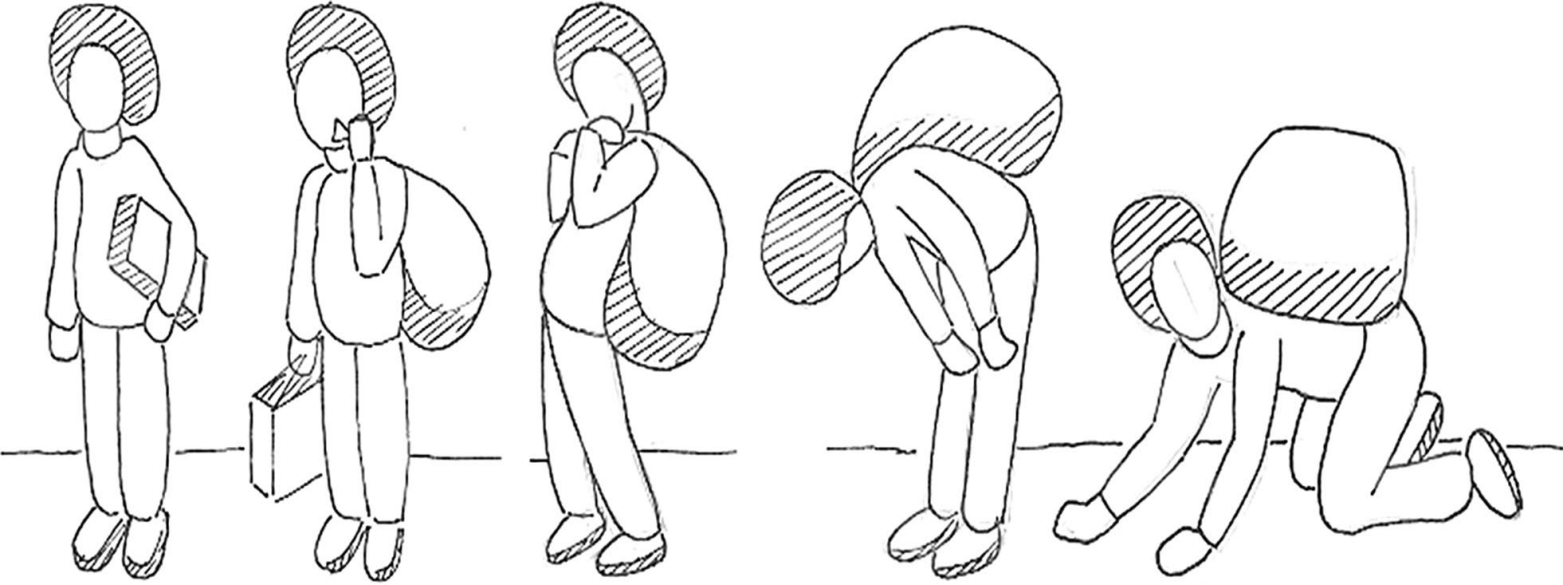
Illustrations of response options for Function scale items

Again, the response options were illustrated, as shown in Fig. 2.

If the respondent indicated no more difficulty in doing a task, the interviewer would go to the next task. If the respondent indicated some degree of difficulty the interviewer asked what caused this difficulty and wrote down the response before going to the next task.

#### Cognitive interviewing

The Function scale was included in the cognitive interviewing exercise described above. The items and process of administering the measure was reviewed following the exercise. Some revisions were made to the Krio version of the items but the ten items for men and for women were retained.

### Phase 2: pilot testing

The final Krio version of both measures was used to collect pilot data in Western Area (urban and rural) and rural areas in Bombali district. The draft measures were administered to 202 respondents (101 female and 101 male) who ranged in age from 18 to 86 (mean age = 39.4, standard deviation = 15.3) and were based in Bombali (100) and Western Area (102).

In addition to the SLPD and Function scale questions, respondents were asked to rate how they felt their life was overall at the moment. They used the illustrations on the Function scale card to do so; they were asked to choose a picture which most closely represented the level of life-difficulties they were currently experiencing. The quantitative data were entered into an Excel spreadsheet, and subsequently into IBM SPSS Statistics for Windows, version 22 for analysis.

Following the completion of the data collection, the research team met to review the process and to discuss the data collectors’ observations of responses to the items. These observations were taken into account when deciding which items to exclude, as well as interpreting results of the statistical analysis.

The performance of each item within the SLPD scale was assessed through the following analyses:

1. Endorsement frequency of items.
2. Discrimination function of items. This was assessed by comparing the responses to items of respondents who rated their life has having no or very few difficulties and those who said they were currently facing a lot or an extreme level of difficulties. It would be anticipated that items capturing significant psychological distress are more likely to be endorsed by those facing higher levels of difficulties.
3. Inter-item correlations (Pearson’s r)—the extent to which items on a scale are assessing the same content. It is unnecessary to have two items measuring the same issue. A correlation between items higher than 0.5 was considered to be high.
4. Internal consistency (Cronbach’s alpha)—how well each item correlates with other items and the total score.
5. Factor analysis—any items which did not load highly onto the factors extracted would be considered for removal.

Retention or removal of items was based on the comprehensive pattern of results, plus feedback from the field researchers following the pilot data collection.

#### Function scale

For both male and female Function scales, descriptive statistics were reviewed, along with field researchers’ feedback on respondents’ reactions to each item.

### Phase 3: validation

The 25-item SLPDS and the 9-item Function scale (male and female versions) were administered to 904 respondents. In addition to the SLPDS and function items, questions were also included on demographic variables and participants’ circumstances and experiences of potentially distressing events.

#### Sampling and data collection

Five districts were purposively selected (Kailahun, Bo, Kono, Kambia, and Western Area) to represent distinct regions of Sierra Leone, and five chiefdoms were selected within each district using a Probability Proportional to Size (PPS) strategy [32]. Within each chiefdom, six villages were randomly selected, and in each village the data collectors surveyed six households that were selected using a “random walk” strategy [33]. The starting points for the random walk for each team of enumerators were selected randomly on a daily basis. Each team selected one card from a pile of folded cards with the five starting points: (1) mosque/church, (2) market/shops, (3) the first house in the entrance to village/section in the urban area, (4) village chief’s house in the rural area or health centre in the urban area, and (5) centre of the village/section for urban settings. To select individuals within the households, we utilised a grid by De Vaus [34]. The enumerators first made a list of individuals (over 18 years old) in a given household who were eligible for the survey from eldest to youngest and assigned a number from 1 to N. Then using the grid, enumerators selected a person based on the order number of the household that the enumerator was surveying for that day and the number of eligible people in the household. Data were collected electronically using tablets programmed with Open Data Kit.

While a minimum of 10 participants per scale item is a widely-used guide for determining the sample size for factor analyses [35], it has been suggested that variable-to-factors ratio and communality between scale items are more important criteria in determining the sample size [36–39]. We followed Mundfrom, Shaw & Ke [39] in estimating a variables-to-factors ratio of four, wide communality (between 0.2 and 0.8) and excellent coefficient congruence (K value 0.98) in gauging target sample size. We opted for wide communality as a middle ground to allow for unexpected trends in data. With the application of these criteria, our target sample size was 900 [39]. Anticipating non-response, we targeted 1100 households. In practice, our data collectors approached 1344 households, 904 of which agreed to take part in the survey.

Our analysis confirmed this sample size to be adequate for factor analysis. The Kaiser–Meyer–Olkin measure of sampling adequacy was 0.953 (above the recommended 0.6) and Bartlett’s test of sphericity was significant (χ^2^ (300) = 7346,5, p < 0.001). Finally, Principal Component Analysis indicated that the communalities were between 0.3 and 0.6, serving as an additional indicator of suitability for factor analysis.

#### Analysis: function scale

The internal reliability of the male and female versions of the scale was estimated using Cronbach’s alpha coefficient, with alpha equal or greater than 0.70 considered satisfactory. Item analysis was also conducted, consisting of the mean and standard deviation of each item, and Cronbach’s alpha if this item was removed.

## Results

### Phase 2: pilot testing

Respondents’ ratings of how they felt their life was overall at the moment are summarised in Table 1 below.

**Table 1:** Rating of overall quality of life at time of interview

Rating of life at the moment	N	%
No difficulty	43	21.3
A little difficulty	78	38.6
Some difficulty	37	18.3
A lot of difficulty	26	12.9
Extremely difficult	18	8.9
Total	202	100

#### SLPD scale

The item analysis of the 38 items included in the pilot version of the SLPD scale found that no items had a high endorsement rate (indicating that a high proportion of respondents experienced this ‘very much’) but nine had low endorsement rates (indicating that a high proportion experienced this ‘not at all’). Some of these items asked about socially undesirable behaviours (e.g. use of abusive language), and others were reported by field researchers as having been difficult to understand (e.g. ‘Have you felt bad about yourself?’). Six items were found to have poor discriminant validity, and six items either correlated with the total score poorly (less than 0.3) or led to an increased reliability (Cronbach’s alpha) if removed. Factor analysis identified three factors with an eigenvalue greater than 2 (ten with an eigenvalue greater than 1), and seven items did not load highly (i.e. greater than 0.4) onto any of the three main factors (varimax rotation).

Retention or removal of items was based on the comprehensive pattern of results, plus feedback from the field researchers following the pilot data collection. This process resulted in a reduction from 38 items to 25.

The internal reliability of the 25-item scale was found to be good (Cronbach’s alpha = 0.91), and inter-item correlations ranged from 0.14 to 0.51, with an average of 0.31, falling within the advised range of 0.20 to 0.40. The scale was able to distinguish between those with less severe and more severe problems (discriminant validity), as shown in Table 2 below.

**Table 2:** Comparison of scores on new scale between those with high and low levels of life-difficulties

Rating of life in general	N	Mean score on new scale	Standard deviation	Comparison of means
No difficulty/a little difficulty	117	12.85	9.28	t (df = 190) = -8.52, p < .001
Moderate difficulty/a lot of difficulties/ extremely difficult	75	26.45	12.80	

#### Function scale

For both male and female Function scales, one item was removed due to a very low endorsement rate: in the male scale, ‘Being respectful to other community members and being kind to them’ and in the female scale, ‘Being helpful to others (especially when they are in dire need)’. The final version of both the male and female versions of the scale, therefore, consisted of questions about nine tasks. The English versions of these tasks are summarised in Table 3 below. Internal reliability was calculated for the two 9-item scales based on the pilot data, and was found to be acceptable (with a Cronbach’s alph of 0.73 for the male scale and 0.80 for the female scale).

**Table 3:** Items included in male and female versions of the Function scale

Women	Male
1. Cooking for the family 2. Making a living (e.g. trading or job) 3. Laundry 4. Cleaning the house 5. Caring for your family (children, parents, husband) 6. Cleaning the community 7. Cooking for community activities 8. Taking care of yourself (like washing regularly and braiding your hair) 9. Praying	1. Taking care of the family (ensuring their safety, guiding the children and spending time with them) 2. Making a living (like trading, farming, commercial bike riding and building and construction) 3. Helping with difficult household work (like weeding grass, splitting wood) 4. Take part in community work and other community programmes (like cleaning, going to meetings) 5. Take part in community occasions like marriage or funeral 6. Ensuring community protection and security 7. Taking care of yourself (like taking bath regularly and cutting hair) 8. Praying 9. Doing things that you enjoy when you have time (playing football, watching games or movies or visiting loved ones)

### Phase 3: validation

#### Description of study sample

A total of 904 respondents were included in the study. Descriptive statistics are shown in Table 4

**Table 4:** Descriptive characteristics of respondents (N = 904)

Characteristic	Sample/n (%)	Mean/ score (SD)
Age in years		40.3 (16.5)
Gender: women	454 (50.0)	
Employment status: any job/ work paid in cash or kind	534 (59.-)	
Education		
None or nursery	392 (43.4)	
Primary only	140 (15.5)	
Above primary	372 (41.1)	
Ethnic group		
Mende	327 (36.2)	
Temne	154 (17.0)	
Kono	145 (16.0)	
Other (9 groups plus ‘other’)	278 (30.7)	
Household size (number of people living in same household)		7.5 (4.02)

#### SLPDS: exploratory factor analysis (EFA) and scale reduction

**Fig. 3:**
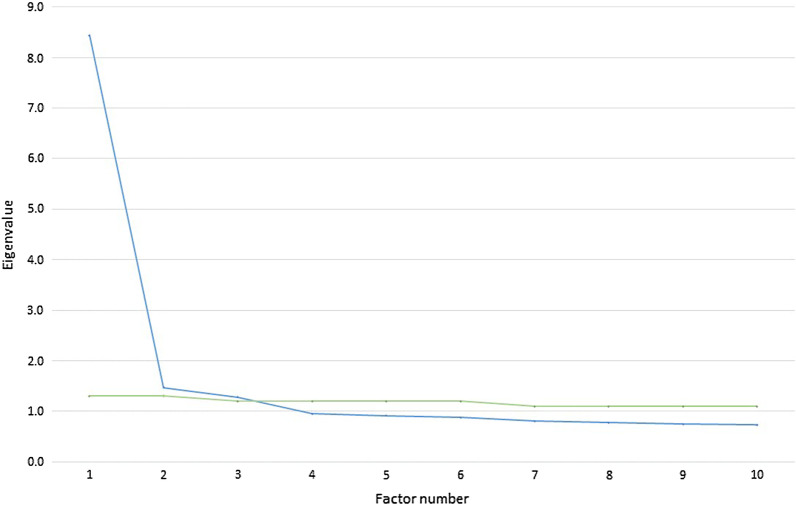
Comparison of study eigenvalues with simulated eigenvalues depicting 3 factor solutions. In parallel analysis, the resulting set of eigenvalues generated from statistically simulated set of random data are averaged and compared with the eigenvalues extracted from the real data. The eigenvalues extracted from real data that exceed those extracted from simulated data indicate the number of factors to retain

Initially, principal component analysis was conducted on the 25 items of the SLPD scale to identify the optimal number of factors to parsimoniously explain the order and structure of items in the scale. Three factors with an eigenvalue greater than 1.0 [40] were retained. Solutions for one, two or three factors were examined using principal axis factoring of the factor loading matrix. A total of 44.8% of the variance was explained by a 3-factor solution. To further identify potential meaningful factors the scree plot was examined. Inspection of Cattell’s scree test (i.e., levelling off of eigenvalues after an “elbow”) supported the appropriateness of a-3 factor solution. This was additionally confirmed by the results of a parallel analysis [41] in which each eigenvalue was compared against an eigenvalue for the corresponding factor in 1000 randomly generated datasets that have similar characteristics to the sample being analysed (Fig. 3). Due to high correlations between 3 factors (r ≥ 0.6), the oblique rotation (promax with the default Kappa 4) was used to achieve rotated factor loadings. For each factor extracted, only individual items with primary loadings at 0.4 or more and no cross-loading at 0.3 and above were retained. The internal consistency of the scale was estimated using Cronbachs' alpha coefficient. Cronbachs' alpha equal or greater than 0.70 was considered satisfactory. Additionally, at each of these steps, item-item bivariate correlations were checked to ensure that items which had very small (r < 0.2) or high correlations (r > 0.5) were removed.

EFA, the Kaiser criterion, the derived scree plot elbow-point and parallel analysis of the 25-item SLPD scale all supported the extraction of 3 factors. EFA with an oblimin rotation for a 3-factor solution (see Additional file 1: Table S1) resulted in the elimination of items 3, 6, 8, 10, 20, 23 and 24. Item 6 ‘Have you forgotten to do things?’ and item 10 ‘Have you felt lonely?’ did not load above 0.3 on any of the factors. Item 20 ‘Have you felt ashamed?’ had loading between 0.3 and 0.4 on both factor 1 and 2. Item 3, 8, 23 and 24 had low loadings (< 0.4) on any of the factors. Table 5 presents the final results for EFA.

**Table 5:** Final results of exploratory factor analysis

	Factors		
	1	2	3
Item 1: Have you had a poor appetite?			0.57
Item 2: Have you preferred to be alone?			0.47
Item 4: Have you felt frustrated?			0.43
Item 5: Have you felt sad?			0.47
Item 7: Have you had difficulty in falling asleep or sleeping well?			0.59
Item 9: Have you been so stressed that you have been moving around a lot?	0.52		
Item 11: Have you felt worried and afraid of what will happen?	0.56		
Item 12: Have you found that you feel happy, then very quickly feel annoyed or sad?	0.75		
Item 13:Have you felt discouraged?		0.55	
Item 14: Have you felt that you are a failure or have let yourself down?		0.52	
Item15: Have you spent a lot of time thinking about your life?	0.52		
Item 16: Have you felt afraid?	0.53		
Item 17: Have you felt confused?	0.66		
Item 18: Have you stopped doing your normal activities (for example work, farming or college) because you don't feel able to continue?	0.47		
Item 19: Have you lost your temper over small things?	0.40		
Item 21: Have you avoided talking to people?		0.71	
Item 22: Have you felt hopeless?		0.77	
Item 25: Have you felt tired of living		0.61	

Extraction method: principal axis factoring; rotation method: promax with kaiser normalization

Internal consistency for the final SLPD scale and 3 subscales was examined using Cronbach’s alpha. Overall, all sub-scales showed good internal consistency with Cronbach’s alpha greater than 0.7 (Table 6). Cronbach’s alpha for the final SLDP scale was 0.89, and no substantial increases in alpha for the scale have been achieved by eliminating more items (Additional file 1: Table S2).

**Table 6:** Item statistics and reliability of the 3 subscales

	Factor 1 (µ ± SD)	Factor 2 (µ ± SD)	Factor 3 (µ ± SD)	SLPD scale (µ ± SD)
Item 9: Have you been so stressed that you have been moving around a lot?	0.62 (0.91)			0.62 (0.91)
Item 11: Have you felt worried and afraid of what will happen?	1.02 (0.99)			1.0 (0.99)
Item 12: Have you found that you feel happy, then very quickly feel annoyed or sad?	1.05 (0.92)			1.1 (0.92)
Item 15: Have you spent a lot of time thinking about your life?	1.55 (1.0)			1.55 (1.0)
Item 16: Have you felt afraid?	0.69 (0.92)			0.69 (0.92)
Item 17: Have you felt confused?	0.90 (0.96)			0.9 (0.96)
Item 18: Have you stopped doing your normal activities (for example work, farming or college) because you don't feel able to continue?	0.71 (0.91)			0.71 (0.91)
Item19: Have you lost your temper over small things?	0.94 (0.97)			0.94 (0.97)
Item 13:Have you felt discouraged?		0.59 (0.87)		0.59 (0.87)
Item 14: Have you felt that you are a failure or have let yourself down?		0.47 (0.84)		0.47 (0.84)
Item 21: Have you avoided talking to people?		0.23 (0.62)		0.23 (0.62)
Item 22: Have you felt hopeless?		0.35 (0.73)		0.35 (0.73)
Item 25: Have you felt tired of living		0.22 (0.62)		0.22 (0.62)
Item 1: Have you had a poor appetite?			0.62 (0.91)	0.62 (0.91)
Item 2: Have you preferred to be alone?			0.39 (0.75)	0.39 (0.75)
Item 4: Have you felt frustrated?			0.74 (0.93)	0.74 (0.93)
Item5: Have you felt sad?			1.23 (1.0)	1.23 (1.0)
Item 7: Have you had difficulty in falling asleep or sleeping well?			0.85 (1.0)	0.85 (1.0)
Cronbach’s alpha	0.81	0.81	0.73	0.89

Review of the item loadings on Factor indicates that it is characterised by high emotional arousal, specifically feelings of fear, anxiety and confusion, and labile mood. This high arousal is connected to physical restlessness and being quick to anger, as well as an inability to continue with normal activities (e.g., work). Factor 2 includes more passive signs of distress, specifically hopelessness and feelings of worthlessness, including being tired of living. It is connected to withdrawal from other people. Factor 3 is characterised by changes in behaviours which are typically associated with wellbeing, such as sleep, appetite and engaging with other people, along with feelings of sadness and frustration.

#### Function scale: reliability

The internal reliability of the female version of the 9-item scale was high (Cronbach’s alpha = 0.90). Table 7 shows the item analyses.

**Table 7:** Item analysis for women’s function scale (regardless of cause of difficulty)

Item	Alpha if deleted	Mean	Std Dev	N
Cooking for the family	0.88	0.58	1.31	450
Making a living (e.g. trading or job)	0.88	0.68	1.39	446
Laundry	0.89	0.53	1.25	449
Cleaning the house	0.89	0.25	0.88	451
Caring for your family (children, parents, husband)	0.89	0.52	1.20	449
Cleaning the community	0.88	0.41	1.24	439
Cooking for community activities	0.87	0.41	1.24	430
Taking care of yourself (like washing regularly and braiding your hair)	0.89	0.19	0.74	453
Praying	0.91	0.10	0.57	454

The only item which would increase the internal reliability of the scale if it was removed was ‘praying’. This item also has a very low mean, indicating that few women said they had difficulty with this task.

The internal reliability of the male version of the 9-item scale was acceptable (Cronbach’s alpha = 0.79), although lower than for the women’s scale. Table 8 shows the item analyses.

**Table 8:** Item analysis for men’s function scale (regardless of cause of difficulty)

Item	Alpha if deleted	Mean	Std Dev	N
Taking care of the family (ensuring their safety, guiding the children and spending time with them)	0.77	0.38	0.97	432
Making a living (like trading, farming, commercial bike riding and building and construction)	0.75	0.55	1.23	423
Helping with difficult household work (like weeding grass, splitting wood)	0.76	0.29	0.96	414
Take part in community work and other community programmes (like cleaning, going to meetings)	0.76	0.14	0.69	436
Take part in community occasions like marriage or funeral	0.76	0.13	0.63	447
Ensuring community protection and security	0.75	0.14	0.71	442
Taking care of yourself (like taking bath regularly and cutting hair)	0.77	0.07	0.53	450
Praying	0.80	0.27	0.80	447
Doing things that you enjoy when you have time (playing football, watching games or movies or visiting loved ones)	0.76	0.18	0.74	449

#### Relationships between SLPDS and function scale

Bivariate correlations were calculated between each of the SLPDS sub-scales and the Function scale for men and for women. The results are shown in Table 9.

**Table 9:** Correlations (Pearson) between SLPD scale and sub-scales and function scale

SLPD scale	Female *r* (p)	Male *r* (p)
SLPDS Factor 1	.133 (.007)	.356 (< .001)
SLPDS Factor 2	− .018 (ns)	.302 (< .001)
SLPDS Factor 3	.308 (< .001)	.299 (< .001)
SLPDS total	.167 (.001)	.381 (< .001)

## Discussion

The study described in this paper aimed to develop a culturally-appropriate measure of psychological distress which would enable government bodies and local and international non-governmental organisations in Sierra Leone to reliably and validly assess mental health and psychosocial needs. This is a necessary step in planning effective service provision, and the evaluation of programmes and initiatives designed to improve mental health and psychosocial wellbeing. A reliable 18-item measure of distress, consisting of three subscales, was developed, along with reliable, gender-specific measures of ability to carry out daily tasks. The survey sample of randomly selected households within five districts reflected the geographical, economic and cultural diversity of the country.

### Utility of the SLPDS in the Sierra Leone context

A large proportion of the locally-appropriate measures of mental health which have been developed up to now have attempted to identify and measure clinical disorders or syndromes [11, 15, 20],. The study described here does not follow this pattern; instead it aims to measure ‘signs of distress’ without drawing any conclusions about clinical significance, or trying to equate experiences of distress with clinical diagnoses such as depression, anxiety of post-traumatic stress disorder. This decision is partly made as a result of the first phase of data collection, from which no clear ‘syndromes’ or local disorders emerged [22]. However, it is also heavily influenced by the recent Lancet Commission on Global Mental Health [42], which recognises that ‘the binary approach to the diagnosis of mental disorders—although useful for health professionals—does not adequately reflect the dimensional nature of mental health or the experience of people affected’ (p4).

An emphasis on diagnostic categories potentially limits the capacity for early identification of the onset of psychological problems [43]. A continuum of distress exists, ranging from mild, time-limited distress to chronic, progressive, and severely disabling conditions. In the early stages of a mental disorder, symptoms are often transient, mixed, and reactive to circumstances, and it is only as the condition progresses or persists that the signs and symptoms allow for a diagnosis. Common signs of distress, such as low mood, are associated with more total disability at a population level than diagnostically defined mental disorders [42]. A measure which facilitates the identification of mild, potentially transient, signs of distress is particularly important in a setting such as Sierra Leone, where there are very limited formal mental health services, but potentially a wide range of community-based support systems and actors. Those with milder forms of distress may benefit from support which can be provided at community level (e.g. promoting self-care, connection to social support initiatives, or increased monitoring), and advocacy for more targeted mental health promotion activities. The dimensional concept of mental health lends itself to identifying public policies that promote and protect mental health for all people, irrespective of the presence of a mental disorder, much more than the restrictive concept of dividing people into those who do not have a mental disorder and those who do.

### Understandings of distress in Sierra Leone

During the initial development of the SLPDS, some potential items remained conceptually difficult. In general, respondents were not comfortable with abstract questions about feelings; they wanted to understand the context and cause of the feeling. For example, when asked whether they had felt afraid over the last one week, a common response was to ask what the interviewer thought they should be afraid of. Similarly, with the question about whether they had felt ashamed over the last one week, and about over-thinking. The concept of a feeling which is not necessarily rational or in response to a specific event did not make intuitive sense to many respondents. This seems to reflect a cultural belief that everything happens for a reason. It is also in line with Betancourt’s [44] observation that more concrete items tend to be more easily understood in a scale. Price, Conteh & Esliker [30] also noted that in Krio, psychological constructs are not easily separated from physical ones. A related challenge (also noted by Kaiser et al [21]) is that, particularly when trying to describe relatively abstract concepts, items sometimes became relatively long and difficult to follow.

Some items were interpreted differently from how they were intended (also experienced by Kaiser et al [21]). For example, the item designed to measure rumination or ‘over-thinking’ (‘Have you spent a lot of time thinking about what is happening in your life?’ or in Krio ‘Yu dɔn spɛn plɛnti tɛm de tink bɔt aw tin dɛm de go na yu layf?’) was understood as referring to constructive or solution-focused thinking and planning, and was seen as a positive behaviour. Some saw the question ‘Have you had trouble concentrating?’ (I dɔn at fɔ mek yu fokɔs pan wan tin fɔ lɔŋ?) as referring to the ability to multi-task; again, this was seen as a positive behaviour.

### Limitations

Challenges were initially experienced in the administration of the Function scale. Respondents often struggled to rate how much difficulty they had with a task compared to another man/ woman of their age, and regularly reverted to simply stating how much difficulty they had with a task. There were particular challenges with the item relating to the activities a respondent undertook to earn a living, with respondents commonly referring to the difficulty they had obtaining money rather than the difficulty they had in performing the income-generating activity itself. As a result, during the pilot phase there were inconsistent ratings of the difficulties respondents had performing their daily tasks, especially those related to income-generation. Through discussion, the research team identified strategies to ensure that these questions were asked in a consistent way and that respondents’ understanding was regularly checked and corrected where necessary to ensure that valid data were obtained using the Function scale. Clear and specific guidance will be required to allow others to use this tool effectively.

It should also be noted that the correlations between scores regarding distress and function were lower than reported in some similar studies. The Function scores relate to difficulties experienced regardless of the cause of the difficulties, not only difficulties caused by emotional distress. The correlations between women’s Function scores and the SLPDS scores are much stronger if only those coded as having an ‘emotional’ cause are included, but this is not the case for men. Our future work plans to address these findings regarding the gendered nature of distress and its relation to function.

Initially, we were planning to conduct Confirmatory Factor Analysis (CFA) to verify the factor structure determined by EFA on data coming from a sample with similar characteristics to our original sample. We were unable to advance this work as planned due to the COVID-19 pandemic and restrictions imposed by governments on international travel. Further work, therefore, is needed to verify the three sub-scales proposed in this work.

## Conclusions

This study describes the development and properties of a culturally-appropriate measure of psychological distress in Sierra Leone. The 18-item distress scale consists of three subscales, all of which showed good internal consistency.Two gender-specific measures of ability to carry out daily tasks also have good internal consistency. Together these two measures provide a locally-validated tool which will enable government bodies and local and international non-governmental organisations in Sierra Leone to assess mental health and psychosocial needs. The availability and use of this measure will support effective service provision and the evaluation of programmes and initiatives designed to improve mental health and psychosocial wellbeing.

## Supplementary Information

**Additional file 1.** Exploratory factor analysis statistical details.

## Data Availability

The datasets generated and analysed during the current study are available from the corresponding author on reasonable request.
