# Supplementary material for: The development of a contextually appropriate measure of psychological distress in Sierra Leone
Source: BMC Psychol. 2021 Jul 21;9:108. doi: 10.1186/s40359-021-00610-w (PMC8294262; doi:10.1186/s40359-021-00610-w)
Supplement: Supplementary file 1 — Additional file 1. Exploratory factor analysis statistical details. [file 40359_2021_610_MOESM1_ESM.docx]

**SUPPLEMENTARY MATERIAL**

Table S1. Exploratory factor analysis for the 3 scales

|  | Factor | | |
| --- | --- | --- | --- |
|  | 1 | 2 | 3 |
| Item 1: Have you had a poor appetite? |  |  | 0.59 |
| Item 2: Have you preferred to be alone? |  |  | 0.45 |
| Item 3: Have you cried easily? |  |  | 0.32 |
| Item 4: Have you felt frustrated? |  |  | 0.45 |
| Item 5: Have you felt sad? | 0.34 |  | 0.48 |
| Item 6: Have you forgotten to do things? |  |  |  |
| Item 7: Have you had difficulty in falling asleep or sleeping well? |  |  | 0.58 |
| Item 8: Have you believed that you were bewitched? |  | 0.39 |  |
| Item 9: Have you been so stressed that you have been moving around a lot? | 0.52 |  |  |
| Item 10: Have you felt lonely? |  |  |  |
| Item 11: Have you felt worried and afraid of what will happen? | 0.55 |  |  |
| Item 12: Have you found that you feel happy, then very quickly feel annoyed or sad? | 0.76 |  |  |
| Item 13: Have you felt discouraged? |  | 0.56 |  |
| Item 14: Have you felt that you are a failure or have let yourself down? | 0.31 | 0.53 |  |
| Item 15: Have you spent a lot of time thinking about your life? | 0.54 |  |  |
| Item 16: Have you felt afraid? | 0.55 |  |  |
| Item 17: Have you felt confused? | 0.65 |  |  |
| Item 18: Have you stopped doing your normal activities (for example work, farming or college) because you don't feel able to continue? | 0.45 |  |  |
| Item 19: Have you lost your temper over small things? | 0.40 |  |  |
| Item 20: Have you felt ashamed? | 0.35 | 0.37 |  |
| Item 21: Have you avoided talking to people? |  | 0.73 |  |
| Item 22: Have you felt hopeless? |  | 0.79 |  |
| Item 23: Have you felt that people dislike you? |  | 0.38 |  |
| Item 24: Have you talked to yourself? | 0.31 |  |  |
| Item 25: Have you felt tired of living |  | 0.65 |  |

*Extraction Method: Principal Axis Factoring.*

*Rotation Method: Promax with Kaiser Normalization.*

*Note. Small factor loadings < 0.3 were suppressed so that results are easier to interpret.*

*Item 6 and item 10 did not load above 0.3 on any of the factors.*

Table S2. Item-Total Statistics

|  | Scale Mean if Item Deleted | Scale Variance if Item Deleted | Corrected Item-Total Correlation | Squared Multiple Correlation | Cronbach's Alpha if Item Deleted |
| --- | --- | --- | --- | --- | --- |
| Item 1 | 12.54 | 81.5 | 0.41 | 0.24 | 0.88 |
| Item 2 | 12.77 | 83.1 | 0.39 | 0.23 | 0.88 |
| Item 4 | 12.42 | 78.6 | 0.58 | 0.39 | 0.88 |
| Item 5 | 11.93 | 77.6 | 0.59 | 0.41 | 0.88 |
| Item 7 | 12.31 | 79.2 | 0.49 | 0.32 | 0.88 |
| Item 9 | 12.54 | 79.6 | 0.53 | 0.32 | 0.88 |
| Item 11 | 12.14 | 77.3 | 0.6 | 0.41 | 0.88 |
| Item 12 | 12.11 | 79.2 | 0.55 | 0.37 | 0.88 |
| Item 13 | 12.57 | 79.3 | 0.58 | 0.42 | 0.88 |
| Item 14 | 12.69 | 79.7 | 0.58 | 0.42 | 0.88 |
| Item 15 | 11.62 | 79.8 | 0.46 | 0.25 | 0.89 |
| Item 16 | 12.47 | 80.2 | 0.48 | 0.29 | 0.89 |
| Item 17 | 12.26 | 77.2 | 0.65 | 0.45 | 0.88 |
| Item 18 | 12.45 | 79.8 | 0.52 | 0.31 | 0.88 |
| Item 19 | 12.22 | 79.8 | 0.48 | 0.27 | 0.89 |
| Item 21 | 12.93 | 82.9 | 0.50 | 0.39 | 0.89 |
| Item 22 | 12.82 | 81.6 | 0.53 | 0.44 | 0.88 |
| Item 25 | 12.94 | 82.7 | 0.53 | 0.38 | 0.88 |
